# Dexmedetomidine as an adjunct to local anesthetics in nerve block relieved pain more effectively after TKA: a meta-analysis of randomized controlled trials

**DOI:** 10.1186/s13018-020-02105-7

**Published:** 2020-12-01

**Authors:** Liping Pan, Hao Wu, Heng Liu, Xin Yang, Zhichao Meng, Yongping Cao

**Affiliations:** Department of Orthopedics, Peking University First Hospital, No. 8 Xishiku Street, XiCheng District, Beijing, 100034 People’s Republic of China

**Keywords:** Dexmedetomidine, Nerve block, Analgesia, TKA

## Abstract

**Background:**

Dexmedetomidine has shown potential in pain control in patients undergoing total knee arthroplasty (TKA). However, the combination of nerve block and dexmedetomidine may be a preferred alternative for postoperative analgesia after TKA. The aim of this study was to perform a meta-analysis on existing randomized controlled trials (RCTs) to determine the efficacy and safety of dexmedetomidine as an adjunct to local anesthetics in nerve block after TKA.

**Methods:**

A literature survey was conducted in the databases of PubMed, Embase, Cochrane Library, Web of science, and ScienceDirect for the RCTs completed before February 1st, 2020 that met pre-specified inclusion criteria. The primary outcomes included the pain scores, duration of analgesia, opioid consumption within 24 h postoperatively, and the level of patient satisfaction. The secondary outcomes included the motor strength, degree of sedation, postoperative nausea and vomiting, and other related complications. The methodological quality was assessed by the Cochrane risk of bias tool.

**Results:**

The initial literature search yielded 143 studies, out of which seven studies met the inclusion criteria. The pooled data indicated that dexmedetomidine combined with local anesthetics in nerve block in TKA decreased the postoperative pain scores at rest as well as at motion (SMD = − 1.01 [95% CI − 1.29 to − 0.72], *p* < 0.01; SMD = − 1.01 [− 1.25 to − 0.77], *p* < 0.01) respectively, decreased the total opioid consumption within 24 h (SMD = − 0.63 [− 0.86 to − 0.40], *p* < 0.01), prolonged the duration of analgesia (SMD = 0.90 [0.64 to 1.17], *p* < 0.01), improved motor strength (SMD = 0.23 [0.01 to 0.45], *p* = 0.04), improved the degree of sedation (SMD = 0.94 [0.70 to 1.18], *p* < 0.01), and increased the level of patient satisfaction (SMD = 0.88 [0.60 to 1.17], *p* < 0.01) without increasing nausea and vomiting (RD = − 0.05 [− 0.11 to 0.01], *p* = 0.14), as well as other complications (RD = − 0.01 [− 0.08 to 0.07], *p* = 0.89), compared with local anesthetics alone.

**Conclusions:**

It is effective and safe for dexmedetomidine as an adjunct to local anesthetics in nerve block in TKA to relieve postoperative pain, decrease total opioid consumption, prolong analgesic duration, and increase patient satisfaction without increasing related complications. Based on the quality of evidence, this meta-analysis recommends that dexmedetomidine can be used in a regular treatment regimen and as an adjunct addition to local anesthetics in nerve block for patients undergoing TKA.

**Registration:**

This meta-analysis was prospectively registered on PROSPERO (International prospective register of systematic reviews) and the registering number was CRD42020169171.

**Supplementary Information:**

The online version contains supplementary material available at 10.1186/s13018-020-02105-7.

## Background

Total knee arthroplasty (TKA) can be considered the most commonly and successfully performed orthopedic surgical procedure for end-stage knee osteoarthritis and rheumatoid arthritis. However, intolerable postoperative pain is one of the most common and frustrating complications that arise for both patients and surgeons. Previous studies have found that postoperative pain after TKA can affect the early phase of rehabilitation and also the psychological state, which can delay patient discharge and early rehabilitation, and cause a heavy economic burden [1, 2]. Therefore, adequate pain management is very important to reduce morbidity and promote the recovery rate after TKA [3].

Nerve blocks such as femoral nerve block, adductor canal block, and epidural block have been more prevalent in TKA postoperative analgesia due to their effectiveness, easy manipulation, and low rate of complications. Local anesthetics such as ropivacaine or bupivacaine have been commonly used in nerve blocks. However, the postoperative analgesic effects and duration of local anesthetics are not good enough, and sometimes have led to delayed ambulation and an increased risk of falling after TKA [4, 5]. To overcome these shortcomings and further improve the analgesic effect, additional endeavors should be devoted to exploring new and effective agents for nerve block.

Dexmedetomidine is a highly selective, specific, and potent α-2 adrenergic receptor agonist that has sedative, anxiolytic, analgesic, anti-hypertensive, and sympatholytic properties [6]. Many surveys had been conducted for its analgesic effect in nerve block. However, it is controversial on the postoperative analgesic effect of dexmedetomidine in nerve block after TKA. Thus, a meta-analysis of randomized controlled trials was conducted to evaluate the efficacy and safety of dexmedetomidine as an adjunct to local anesthetics in nerve block after TKA.

## Methods

### Search strategy

A systematic and thorough search of medical literature was performed through such repositories as PubMed (Medline), Embase, and the Cochrane Library from inception to February 1st, 2020. Additional searching was conducted in the Web of science and Science Direct databases. The applied searching string was “dexmedetomidine” and “block” and (“knee arthroplasty” or “replacement”).

### Inclusion criteria

The following inclusion criteria in regard to population, intervention, comparator, outcomes, and study design were taken into consideration to select the studies from literature. Population: patients scheduled for primary TKA. Intervention: patients in which nerve block was given using dexmedetomidine combined with regional anesthetics. Comparator: patients in which nerve block was given using regional anesthetics alone. Outcomes: The primary outcomes included pain scores (including the VAS and the NRS) at rest and movement at different points of postoperative time, the total opioid consumption within 24 h postoperatively, the analgesic duration time, and level of patient satisfaction. Secondary outcomes included motor strength, degree of sedation, and the incidence rate of complications, such as nausea, vomiting, bradycardia, and others. Study design: interventional studies. Only published randomized controlled trials (RCTs) in the English language were included. Only those studies that contained a minimum of one outcome were included. The studies must have a follow-up rate of at least 80%. Exclusion criteria included observational studies, non-RCTs, review articles, and studies unpublished or in progress.

### Data extraction

Two independent investigators reviewed the studies and extracted the data. Any discrepancy between the extracted data was resolved by consensus. For each publication, the following information was extracted: first author’s name, year of publication, study location, sample size, study design, gender, population, age, interventions provided, dosages and type of nerve block, the randomization, and blindness processes of the RCTs. In some cases, the corresponding authors of the included RCTs were also contacted to obtain any missing data.

### Risk of bias and quality assessment

The quality assessment of the studies was based on the Cochrane Handbook for Systematic Reviews of Interventions [7]. The parameters of the included literature, such as random sequence generation, allocation concealment, blinding of participants and personnel, blinding of outcome assessment, incomplete outcome data, selective reporting, and other biases were utilized for the assessment of quality.

### Statistical analysis

Statistical analysis and data synthesis were performed with the Review Manager Software for Windows (version 5.3) to assess the data. The standard mean difference (SMD) was used to assess the continuous outcomes such as pain scores, total opioid consumption, analgesia duration, level of patient satisfaction, motor strength, and degree of sedation with a 95% confidence interval (CI). To assess dichotomous outcomes, the risk difference (RD) with a 95% CI was used. The inverse variance and Mantel-Haenszel methods were used to combine the separate statistics. Statistical heterogeneity of the included studies was evaluated using a chi-square test in accordance with the values of *I*^2^. *I*^2^ < 50% and *I*^2^ > 50% were considered irrelevant and relevant heterogeneity, respectively. A fixed-effects model was applied to conduct the meta-analysis when there was no heterogeneity. Otherwise, a random-effects model was used. *P* values < 0.05 were considered statistically significant.

## Results

### Search result and risk assessment

**Fig. 1:**
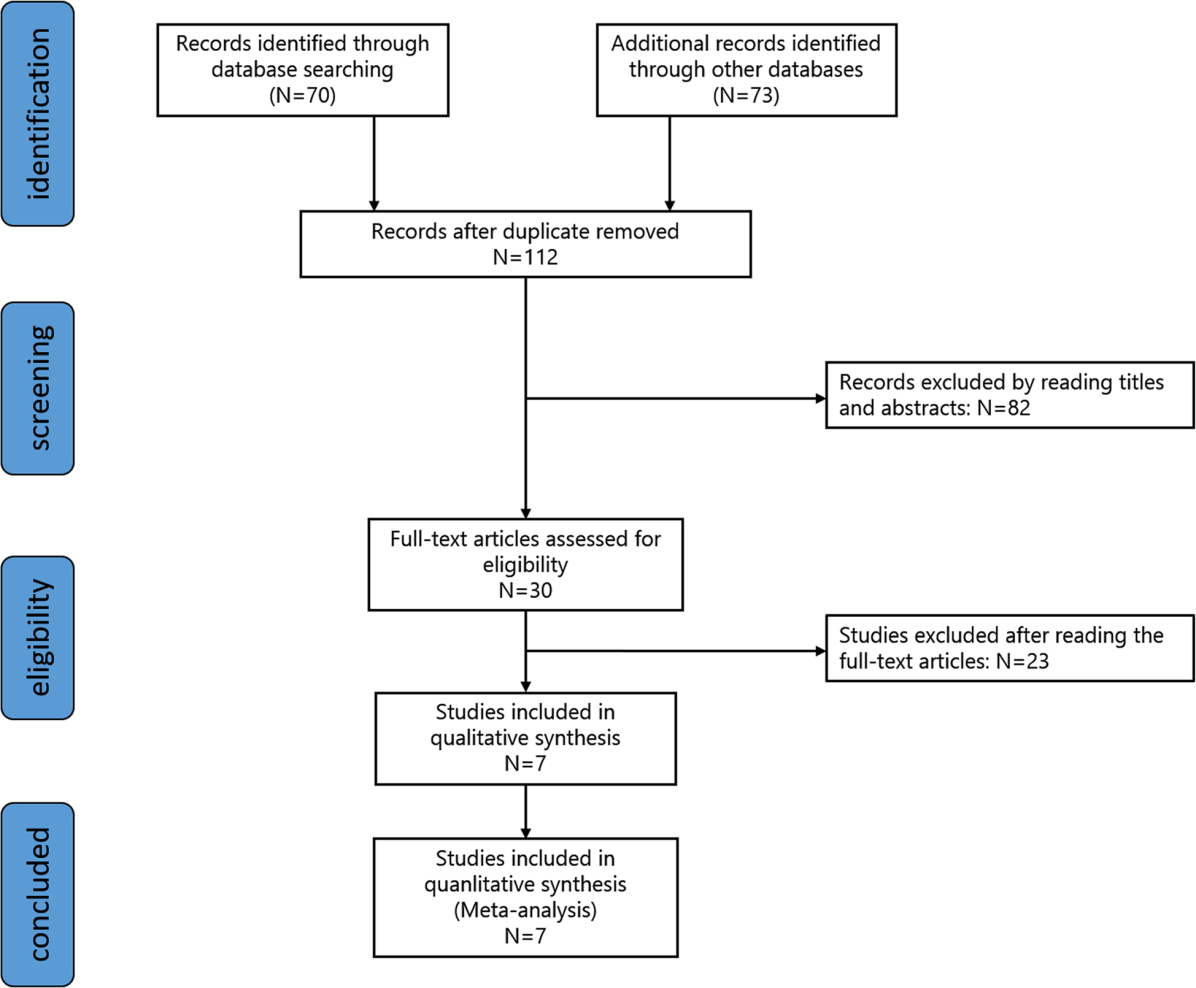
Search results and selection procedure

**Fig. 2:**
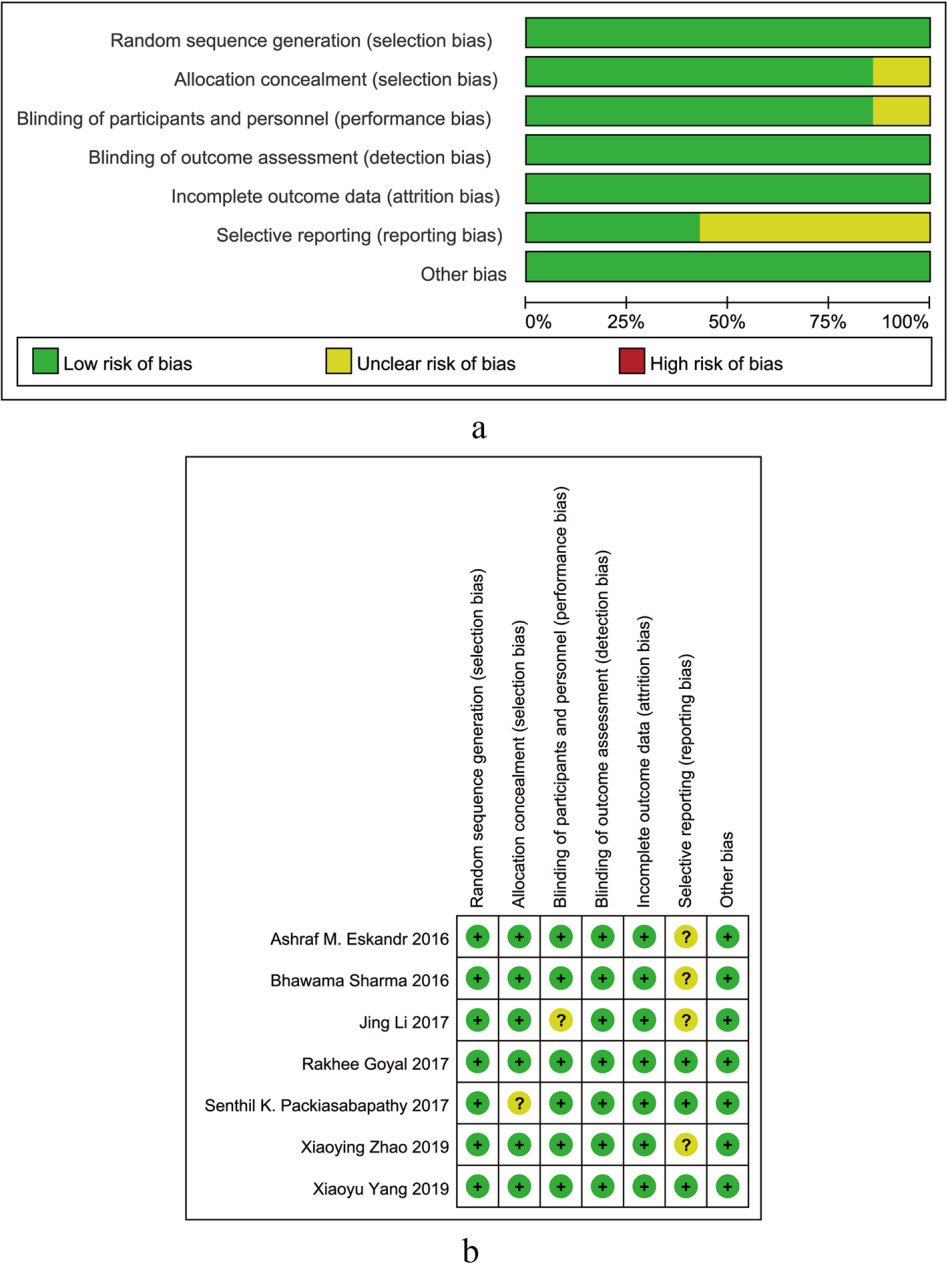
Risk of bias summary: **a** summary of authors’ judgment about each risk of bias item presented as percentages across all included studies; **b** summary of authors’ judgments about each risk of bias item for each included study

The initial literature search identified 143 articles, of which 135 were excluded because they failed to meet the eligibility criteria. A total of 7 RCTs involving 546 participants were ultimately included in this meta-analysis [8–14]. The PRISMA flow diagram is presented in Fig. 1. Four RCTs [8, 10, 12, 14] were considered with an unclear risk of bias due to the lack of availability of the original protocols. One RCT [11] was considered with unclear risk of bias for including no descriptions concerning the concealment of allocation. Another RCT [10] was considered with unclear risk of bias for containing no descriptions about blinding procedures. The results are shown in Fig. 2.

### Study characteristics

Six of 7 RCTs were randomized and double-blinded with adequate allocation concealment except one which was single-blinded. All participants were divided into two main groups. The Dex groups referred to those patients who received dexmedetomidine combined with ropivacaine or bupivacaine in nerve block. The Con groups on the other hand referred to those patients who received only ropivacaine or bupivacaine in nerve block. All the RCTs were conducted since the year of 2016. Five RCTs explored the femoral nerve block, one explored the adductor canal block, and the remaining one explored the epidural nerve block. The detailed characteristics of each RCT are described in Additional file 1.

### Primary outcomes

#### Pain scores

**Fig. 3:**
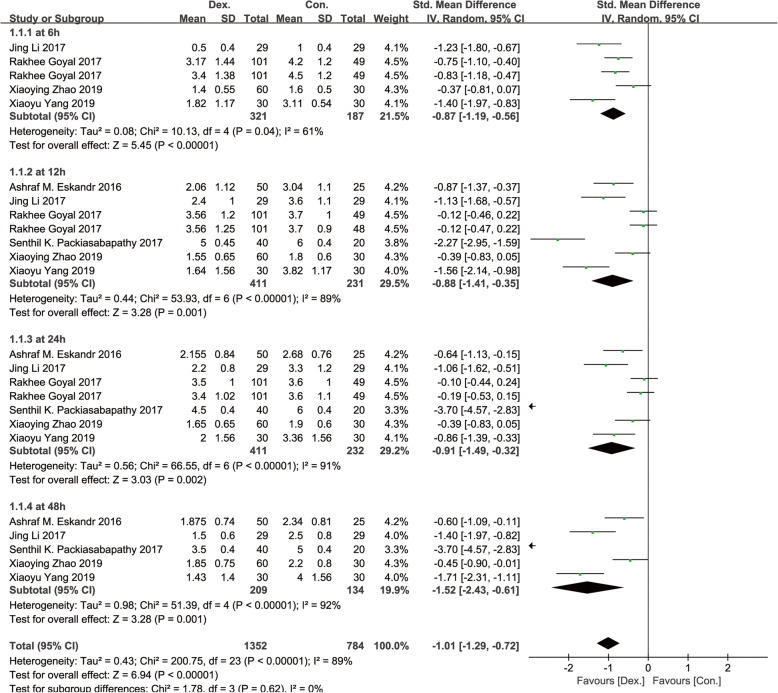
A funnel plot of pain scores at rest at 6, 12, 24, and 48 h postoperatively. Dex: dexmedetomidine combined with local anesthetics; Con: local anesthetics alone

**Fig. 4:**
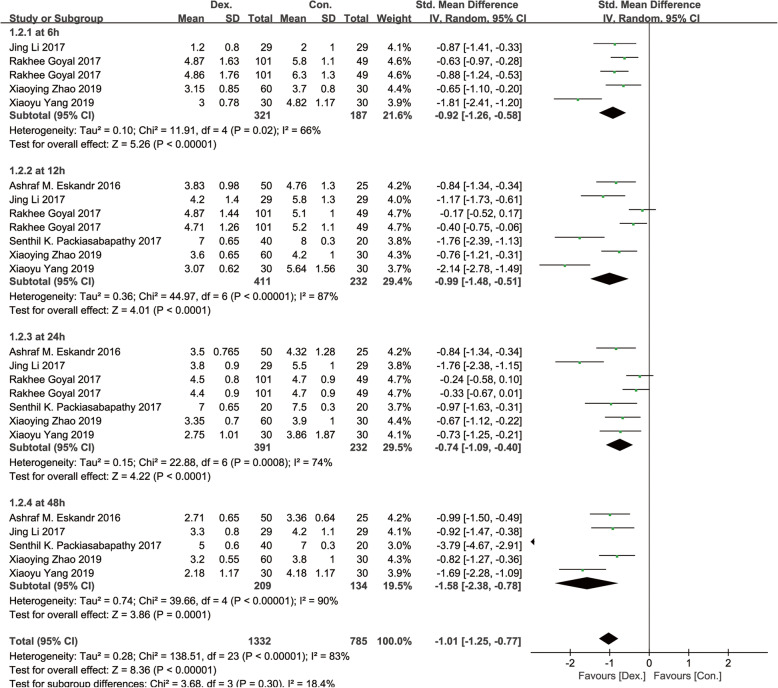
A funnel plot of pain scores at motion at 6, 12, 24, and 48 h postoperatively. Dex: dexmedetomidine combined with local anesthetics; Con: local anesthetics alone

Six studies [8–11, 13, 14], including 496 patients, were reported with visual analogue scale (VAS) or numerical rating scale (NRS) scores at rest and at motion. Significant differences were found in the postoperative pain scores at rest (SMD, − 1.01 [95% CI − 1.29 to − 0.72], *p* < 0.01, *I*^2^ = 89%, Fig. 3) and motion (SMD, − 1.01 [95% CI − 1.25 to − 0.77], *p* < 0.01, *I*^2^ = 83%, Fig. 4) between the Dex and Con groups. Significant differences were also found in the pain scores at rest between the Dex and Con groups at 6 h (SMD = − 0.87 [95% CI − 1.19 to − 0.56], *p* < 0.01, *I*^2^ = 61%), 12 h (SMD = − 0.88 [− 0.41 to − 0.35], *p* < 0.01, *I*^2^ = 89%), 24 h (SMD = − 0.91 [− 1.49 to − 0.32], *p* < 0.01, *I*^2^ = 91%), and 48 h (SMD = − 1.52 [− 2.43 to − 0.61], *p* < 0.01, *I*^2^ = 92%) after surgery. Significant differences were found in the pain scores at motion between the Dex and Con groups at 6 h (SMD = − 0.92 [− 1.26 to − 0.58], *p* < 0.01, *I*^2^ = 66%), 12 h (SMD = − 0.99 [− 1.48 to − 0.51], *p* < 0.01, *I*^2^ = 87%), 24 h (SMD = − 0.74 [− 1.09 to − 0.40], *p* < 0.01, *I*^2^ = 74%), and 48 h (SMD = − 1.58 [− 2.38 to − 0.78], *p* < 0.01, *I*^2^ = 90%) after surgery. A random-effects model was used for the pain scores parameter as significant heterogeneity was found at rest and at motion.

#### Total opioid consumption within 24 h postoperatively

**Fig. 5:**
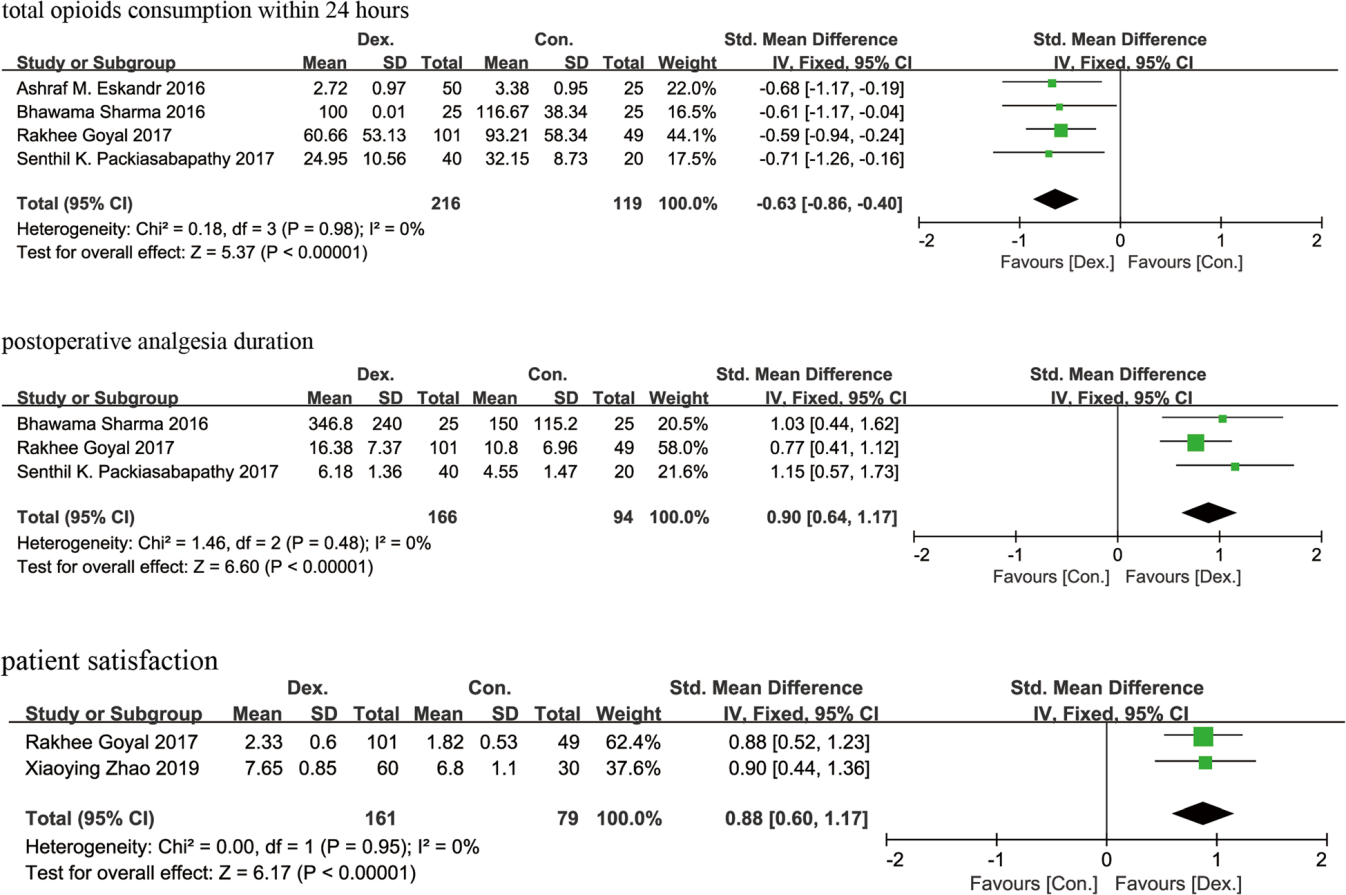
A funnel plot of total opioid consumption within 24 h postoperatively, analgesia duration and patient satisfaction. Dex: dexmedetomidine combined with local anesthetics; Con: local anesthetics alone

Total opioid consumption was recorded in 4 studies [8, 9, 11, 12] containing 336 patients. Pooled data indicated that there were significant differences between the Dex and Con groups (SMD = − 0.63 [− 0.86 to − 0.40], *p* < 0.01, *I*^2^ = 0%, Fig. 5). As no heterogeneity was found between the studies for this parameter, a fixed-effects model was used.

#### Analgesia duration

The duration of analgesia was recorded in 3 studies [9, 11, 12] containing 261 patients. Significant differences were found between the Dex and Con groups (SMD = 0.90 [0.64 to 1.17], *p* < 0.01, *I*^2^ = 0%, Fig. 5). A fixed-effects model was used because no heterogeneity was found between the studies for this parameter.

#### Patient satisfaction

The level of patient satisfaction was recorded in only 2 studies [9, 14] comprising 241 patients. Significant differences were found between the Dex and Con groups (SMD = 0.88 [0.60 to 1.17], *p* < 0.01, *I*^2^ = 0%, Fig. 5). As no heterogeneity was found between the studies, a fixed-effects model was used for this parameter.

### Secondary outcomes

#### Motor strength

**Fig. 6:**
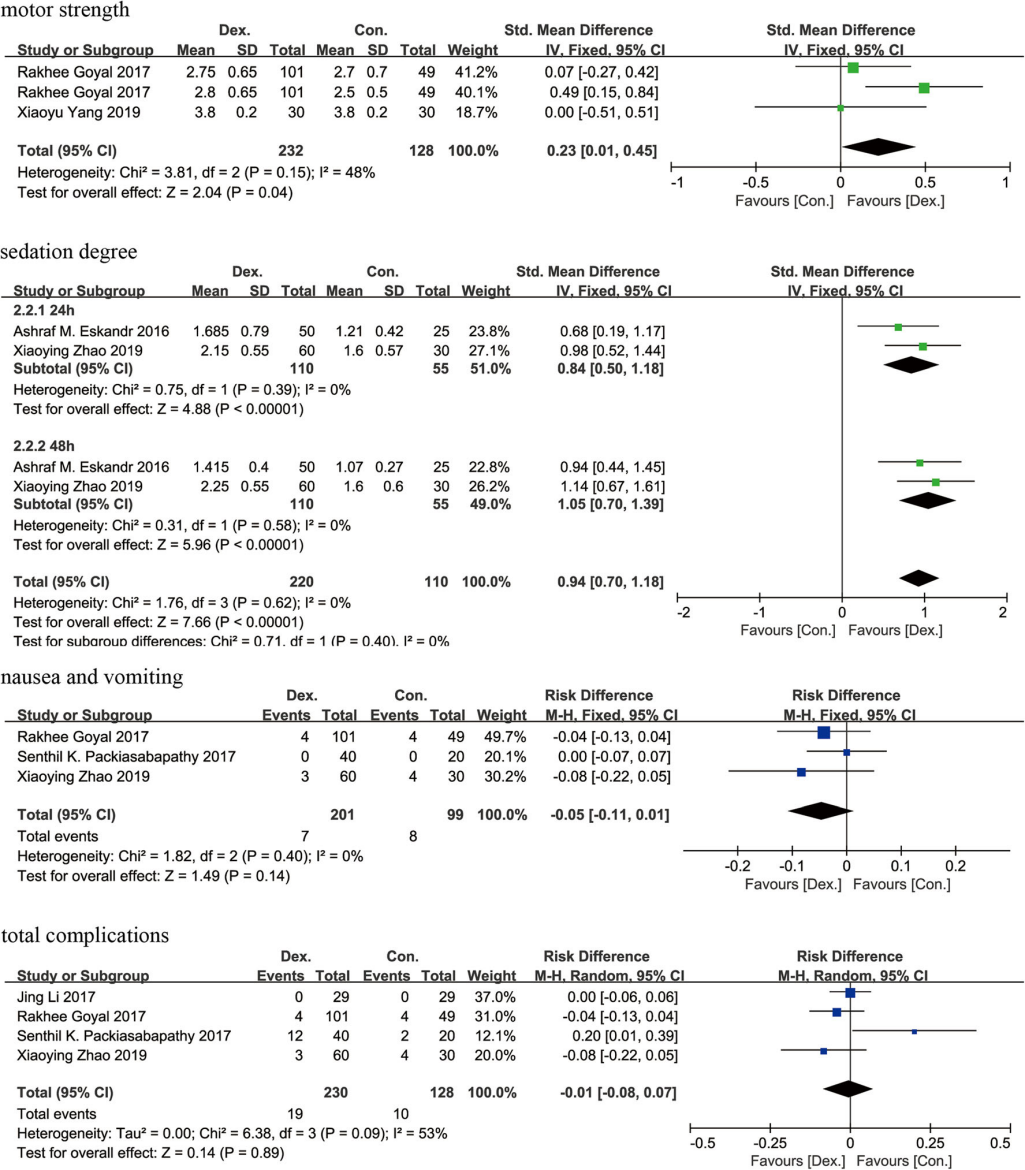
A funnel plot of motor strength, sedation degree, the incidence of postoperative nausea and vomiting and total complications postoperatively. Dex: dexmedetomidine combined with local anesthetics; Con: local anesthetics alone

Motor strength was recorded in 2 studies [9, 13] comprising 211 patients. Significant differences were found between the Dex and Con groups (SMD = 0.23 [0.01 to 0.45], *p* = 0.04, *I*^2^ = 48%, Fig. 6). A fixed-effects model was used because minor heterogeneity was found for this parameter between the studies.

#### Sedation degree

Sedation degree for postoperative 24 and 48 h was recorded in 2 studies [8, 14] comprising 165 patients. Significant differences were found between the Dex and Con groups (SMD = 0.94 [95% CI 0.70 to 1.18], *p* < 0.01, *I*^2^ = 0%, Fig. 6). As no heterogeneity was found between these studies for this parameter, a fixed-effects model was used.

#### Nausea and vomiting

Nausea and vomiting was recorded in 3 studies [9, 11, 14] containing 301 patients. No significant differences were found between the Dex and Con groups (RD = − 0.05 [− 0.11 to 0.01], *p* = 0.14, *I*^2^ = 0%, Fig. 6). As no heterogeneity was found between these studies for this parameter, a fixed-effects model was used.

#### Total complications

Total complications were recorded in 4 studies [9–11, 14] comprising 361 patients. No significant differences were found between the Dex and Con groups (RD = − 0.01 [− 0.08 to 0.07], *p* = 0.89, *I*^2^ = 53%, Fig. 6). A random-effects model was used because median heterogeneity was found for this parameter between the studies.

## Discussion

Nerve block is one of the most common and effective analgesic methods that has been widely used in TKA postoperatively. Local anesthetics such as ropivacacine or bupivacaine are the most commonly used in nerve blocks. Dexmedetomidine, a highly selective, specific, and potent α-2 adrenergic receptor agonist, has beneficial analgesic effects for anesthetic procedures. Several studies [13, 14] have demonstrated that dexmedetomidine combined with local anesthetics can improve the postoperative analgesic effect and further prolong the duration of time after TKA. However, the size of these studies has been very small and the results are also inconsistent and not persuasive. Therefore, the current meta-analysis explored the efficacy and safety of dexmedetomidine as an adjunct to local anesthetics in nerve block after TKA.

According to the GRADE system [15], pooled data with the median-quality evidence of this meta-analysis found that, compared with local anesthetics alone, dexmedetomidine combined with local anesthetics reduced pain scores at rest and at motion postoperatively, reduced total opioid consumption, prolonged the analgesia duration, increased the patient satisfaction level, and improved the sedation without increasing the risk of nausea, vomiting, and other common complications after TKA. Low-quality evidence with limited samples found that this combination also improved motor strength.

The primary outcomes, including the pain score, total opioid consumption, and analgesic duration, were explored with median-quality evidence. Our meta-analysis indicated that dexmedetomidine combined with local anesthetics in nerve block significantly reduced the postoperative pain score at rest and motion, reduced total opioid consumption, and prolonged the duration of analgesia. Qi Yang et al. [16] also found that dexmedetomidine administered mostly in vein reduced the postoperative pain scores with high heterogeneity (*I*^2^ = 90%). Our study also showed that the pain scores significantly decreased at different time points postoperatively in the Dex groups compared with the Con groups even though some heterogeneity was found. The effect tendency between each study was concordant that the pain score in the Dex groups was reduced compared to the Con groups in each and every included study. Some of these studies also found great heterogeneity when exploring the pain scores without proper interpretation [6, 17]. However, the small number of trials precluded sufficient exploration of heterogeneity through subgroup or meta-regression analysis. We found that the total opioid consumption was significantly decreased and that the duration of analgesia was significantly prolonged in the Dex groups as compared with the Con groups. This result further reflected the decreased postoperative pain intensity with no heterogeneity or bias risk. This was consistent with the results in a prospective, randomized, controlled, double-blinded crossover trial conducted in 14 healthy volunteers [18]. However, the underlying mechanism of this prolonged analgesic effect remains unclear because the half-life of dexmedetomidine is only 2 h in tissues. There are different pathways between the sedation and analgesic effects of dexmedetomidine that should be noted [16]. The effect of dexmedetomidine is mediated by the ascending noradrenergic pathway in the locus coeruleus, while the analgesic effect occurs via an α-2 adrenergic receptor-dependent descending pathway in the spinal cord [19]. Animal studies have shown that the addition of dexmedetomidine to local anesthetics can increase the duration of sensory and motor blockades in a rat model of sciatic nerve block [20].

Motor strength was another important factor to keep track of for the patients as well as for the surgeons because early rehabilitation is essential and helpful for the success of TKA. However, some of the studies revealed that the nerve block was associated with delayed ambulation [4] and also a risk for falling [5]. Low quality evidence with a limited sample size in the current meta-analysis showed that dexmedetomidine combined with local anesthetics in nerve block could increase motor strength compared to local anesthetics alone. This is likely due to the fact that dexmedetomidine can inhibit the local anesthetics to penetrate the motor fiber. As we know, ropivacaine is less lipophilic than bupivacaine and less likely to penetrate the large myelinated motor fibers [21]. Perhaps the addition of dexmedetomidine can help the local anesthetics to penetrate the motor fibers less. However, larger RCTs and additional endeavors should be undertaken to confirm and explain this effect.

Median-quality evidence showed a better degree of sedation when dexmedetomidine was combined with local anesthetics in nerve block after TKA. Dexmedetomidine had a good effect on sedation which is commonly used in general anesthesia [6]. So even administered in nerve block, its sedation effect could not be totally eliminated. This could do some help for most of the patients who would feel anxiety for their operation and postoperative pain.

Median-quality evidence also showed a similar incidence of postoperative nausea and vomiting, and other related complications, between the Dex and Con groups. Nausea and vomiting, hypotension, bradycardia, neuropathy, infection, and excessive sedation are possible complications related to dexmedetomidine or nerve block [22–24]. These results in our meta-analysis was consistent with another meta-analysis that explored knee arthroscopy [25]. On the contrary, the meta-analysis by Qi Yang et al. [16] found that dexmedetomidine treatment could decrease the incidence of postoperative nausea and vomiting, and increase the risk of bradycardia in TKA. Our study revealed that it is safe to add dexmedetomidine to nerve block to some extent; however, more attention should be paid to the dosage of dexmedetomidine especially in the elderly patients. Median-quality evidence showed that dexmedetomidine combined with local anesthetics could improve the level of patient satisfaction. Better analgesia, lower opioid consumption, improved ambulation, early rehabilitation, and limited complications are the possible contributory factors for higher level of patient satisfaction.

Our meta-analysis exhibited various limitations that should be noted. Firstly, only 7 RCTs were included in our meta-analysis, and the sample size of every RCT was limited. Therefore, RCTs with larger sample sized has been needed to validate these results. Secondly, the included RCTs only evaluated the immediate effects within 48 h after TKA, and longer measures were not explored and long-term follow-ups has been needed to further investigate the functional outcomes. Thirdly, only English publications were included in our meta-analysis and multi-cultural studies should be included in the future to generalize these results. Fourthly, with regard to the significant heterogeneity of postoperative pain scores, the source of heterogeneity was not determined with proper interpretation. However, the effect tendency between each study was concordant.

## Conclusions

Compared with local anesthetics alone in nerve block, dexmedetomidine combined with local anesthetics can better relieve postoperative pain, decrease the total opioid consumption, prolong the duration of analgesia, and improve patient satisfaction level without any other related postoperative complications. Nevertheless, more prospective RCTs with larger sample sizes are needed to validate the effectiveness and safety of this treatment regimen. When taking the quality of evidence in this meta-analysis into consideration, we recommend that dexmedetomidine should be used as an adjunct to local anesthetics in nerve block after TKA in clinical practice.

## Supplementary Information

**Additional file 1.** characteristics of included studies

## Data Availability

Not applicable.

## Abbreviations

TKA: Total knee arthroplasty
VAS: Visual analogue scale
NRS: Numerical rating scale
PRISMA: Preferred Reporting Items for Systematic Reviews and Meta-Analyses
RCT: Randomized controlled trials
